# Anti-malarial drugs and the prevention of malaria in the population of malaria endemic areas

**DOI:** 10.1186/1475-2875-9-S3-S2

**Published:** 2010-12-13

**Authors:** Brian Greenwood

**Affiliations:** 1 Department of Infectious and Tropical Diseases, London School of Hygiene and Tropical Medicine, Keppel St., London WC1E 7HT, UK

## Abstract

Anti-malarial drugs can make a significant contribution to the control of malaria in endemic areas when used for prevention as well as for treatment. Chemoprophylaxis is effective in preventing deaths and morbidity from malaria, but it is difficult to sustain for prolonged periods, may interfere with the development of naturally acquired immunity and will facilitate the emergence and spread of drug resistant strains if applied to a whole community. However, chemoprophylaxis targeted to groups at high risk, such as pregnant women, or to periods of the year when the risk from malaria is greatest, can be an effective and cost effective malaria control tool and has fewer drawbacks. Intermittent preventive treatment, which involves administration of anti-malarials at fixed time points, usually when a subject is already in contact with the health services, for example attendance at an antenatal or vaccination clinic, is less demanding of resources than chemoprophylaxis and is now recommended for the prevention of malaria in pregnant women and infants resident in areas with medium or high levels of malaria transmission. Intermittent preventive treatment in older children, probably equivalent to targeted chemoprophylaxis, is also highly effective but requires the establishment of a specific delivery system. Recent studies have shown that community volunteers can effectively fill this role. Mass drug administration probably has little role to play in control of mortality and morbidity from malaria but may have an important role in the final stages of an elimination campaign.

## Background

This review on the use of anti-malarial drugs to prevent malaria in the population of malaria endemic areas focuses on Africa where the potential for this approach to malaria control is greatest. Some of the issues reviewed here are relevant to other high transmission areas, such as Papua New Guinea and a few parts of South East Asia but, in general, diagnosis and treatment is a more relevant malaria control strategy in the low to medium transmission areas of Asia and South America than chemoprevention.

**Figure 1:**
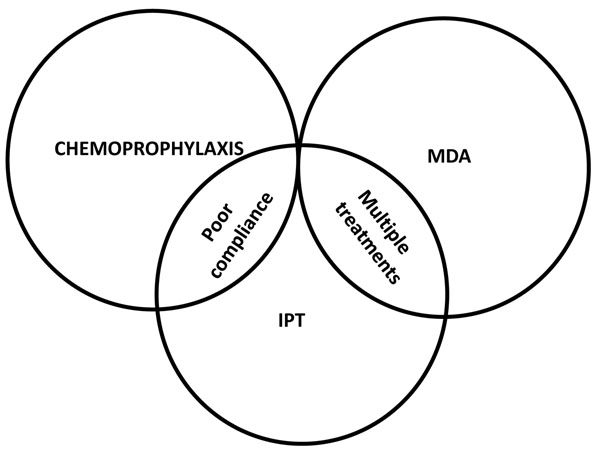
Overlap in the different forms of chemoprevention. Adapted from reference [2].

Anti-malarial drugs can be used to prevent malaria in a number of different ways (Table 1). However, there is considerable overlap between these different approaches (Figure 1) [1]. For example, it is now clear that the major mode of action of Intermittent Preventive Treatment (IPT) in infants (IPTi) and in children (IPTc) is prophylaxis, so that this intervention overlaps with approaches previously termed seasonal or targeted chemoprophylaxis. Furthermore, several mass drug administration (MDA) programmes have involved administration of several rounds of treatment and this approach, therefore, overlaps with IPT.

**Table 1:** Definitions of different forms of chemoprevention.

a. *Chemoprophylaxis.* This term is used to describe the administration of an antimalarial drug or drug combination in such a way that blood levels are maintained above the inhibitory level of survival of the local strains of parasite for the whole of the period at risk and, in the case of travellers, for an appropriate period afterwards in order to kill emerging liver forms.
b. *Intermittent preventive treatment (IPT)*. This term is used to describe the administration of a full curative dose of an antimalarial or antimalarial combination to a selected, target population at specified times without determining whether or not the subject is infected. It is recognized that between drug administrations blood levels may fall below the protective level and that infections may still occur although some of these may be truncated by persistent low levels of drug.

c. *Mass drug administration (MDA).* This term is used to describe the administration of an antimalarial drug or drug combination to a whole population without screening for the presence of infection. MDA may involve either the administration of a full therapeutic course or a sub-therapeutic one as once practized through the use of medicated salt.

## Chemoprophylaxis

Anti-malarial drugs have been used to prevent malaria in travellers to malaria endemic areas for hundreds of years and the use of prophylactic quinine was one of the key features that enabled Europeans to colonize the highly endemic regions of West and Central Africa. Without quinine, death from malaria was almost inevitable. More recently, chemoprophylaxis has been used to protect effectively the millions of tourists who visit malaria endemic areas each year for business or holidays. In view of the efficacy of chemoprophylaxis in protecting non-immunes, it is surprising that so little attention has been given to the use of drugs to protect the local population. If prophylaxis can be used effectively to protect an expatriate and his family for decades why should the same approach not be used to protect at risk groups in the local population?

Because of the increased risk of malaria in pregnancy, chemoprophylaxis with pyrimethamine or chloroquine [CQ] was used quite widely in some parts of Africa in the 1950s and 1960s and, for a while, chemoprophylaxis with CQ was a WHO recommended policy for the prevention of malaria in pregnancy. Chemoprophylaxis with an effective drug reduced maternal anaemia and low birth weight [2], but prophylaxis with CQ fell into disuse because of side effects and increasing resistance to this drug. It was not appreciated at this time that anti-malarials continue to be effective in preventing malaria after resistance has increased to a degree when they are no longer effective for treatment of clinical malaria in children, the group in whom drug sensitivity is usually measured [3].

Only a relatively small number of trials of chemoprophylaxis have been undertaken in children living in endemic areas but these have shown convincingly that chemoprophylaxis with an effective drug decreases deaths from malaria, prevents uncomplicated attacks of malaria, reduces the prevalence of anaemia and improves school attendance [4]. Despite these impressive results, chemoprophylaxis in children has rarely been deployed on a large scale in malaria endemic populations. A few, large commercial concerns have used chemoprophylaxis to protect their work force and their families from malaria and, for a while, a small number of countries, such as Senegal, implemented widespread prophylaxis with CQ in children during the malaria transmission season but this was not sustained.

In view of the results of carefully conducted trials that have demonstrated the efficacy of chemoprophylaxis in preventing mortality and morbidity from malaria in African children why have countries in Africa not adopted this highly effective control measure? There are number of reasons why this has been the case, some of which have more foundation than others. These include -

a. *Cost.* Purchasing and delivering prophylactic drugs to the whole population indefinitely would be costly and so this approach has never been seriously entertained. Instead a focus has been placed on high-risk groups such as pregnant women and young children. Economic evaluation has shown that chemoprophylaxis with a relatively inexpensive drug targeted at pregnant women or children is cost effective [5].

b. *Sustainability*. Sustainability is a challenge for any long-term preventive programme. Several malaria chemoprophylaxis programmes, for example a large programme in Sierra Leone, started well but tailed off due to lack of enthusiasm on the part of the deliverers and the recipients. However, this is not inevitable and delivery of anti-malarials for prevention can be sustained over many years, even when unpaid volunteers are used [6]. Sustainability is being achieved in several mass drug administration programmes such as those being used to control onchocerciasis, filariasis and intestinal helminths.

c. *Acceptability*. The bitter taste of CQ and its propensity to cause itching contributed to the poor uptake of CQ prophylaxis in pregnancy with both pregnant women being reluctant to take the drug on a regular basis and health workers reluctant to provide it. Pyrimethamine was better accepted but is no longer efficacious. Any drug used for prophylaxis in large populations must be well tolerated as well as safe and few anti-malarials meet both of these criteria.

d. *Drug resistance*. Widespread use of drugs for prevention, especially if used in an uncoordinated way, will inevitably increase drug pressure and facilitate the spread of resistant parasites. However, targeting chemoprophylaxis to at risk groups, thus reducing the overall number of parasites in a community exposed to the drug, reduces this risk.

e. *Loss of immunity*. There is some evidence that provision of prophylaxis to young children, in particular to infants, impairs the development of natural immunity and thus increase their susceptibility to malaria when the intervention is stopped [7,8]. However, this is not a problem specific to chemoprevention as this constraint applies to any effective anti-malarial intervention, for example vector control, which is not sustained. However, stopping a sustained period of chemoprevention may pose a greater risk than failing to replace an insecticide treated bed net (ITN) or to continue with indoor residual spraying [IRS] as the transition from protected to unprotected status will occur more rapidly.

Some of the challenges to the use of chemoprophylaxis in the population of endemic countries are soluble. For example, restricting chemoprophylaxis to groups most at risk and/or to the periods of the year when the risk of malaria is highest reduces drug pressure and costs and enhances the chances of sustainability. Development of new, well-tolerated anti-malarials which provide a long period of protection, ideally several months, would reduce the problems of delivery and compliance and might reduce cost even further.

## Intermittent preventive treatment

Intermittent preventive treatment (IPT), probably an inappropriate name as it appears that much of the impact of IPT is achieved through prophylaxis, involves giving a full curative dose of an anti-malarial or anti-malarial combination at fixed times regardless of whether or not a subject is known to be infected. IPT differs from chemoprophylaxis in that drug levels are allowed to fall below protective levels between treatments. Thus, IPT would be expected to be less efficacious than chemoprophylaxis but to have less of a deleterious effect on the development of naturally acquired immunity, by allowing occasional exposure to parasites, and to exert less drug pressure. IPT was investigated initially as a means of controlling malaria in pregnancy but it has subsequently been extended to the prevention of malaria in infants, young children and school children.

## Pregnancy

IPT with a single dose of sulphadoxine/pyrimethamine (SP) was introduced as an approach to the control of malaria in pregnancy because of the unpopularity of CQ chemoprophylaxis and increasing CQ resistance. Early studies showed that IPT, given two or three times during the second and third trimesters of pregnancy, was at least as effective at preventing maternal anaemia and low birth weight as chemoprophylaxis with CQ and much more acceptable [3]. Thus, IPT with SP is recommended by WHO for all pregnant women resident in areas with a moderate or high level of malaria transmission [9]. How IPT in pregnancy (IPTp) with SP achieves its impact is still not fully understood. SP provides 4- 6 weeks prophylaxis against sensitive isolates and thus, if given as recommended on two or three occasions during the second and third trimesters, it will provide prophylaxis for only about half of this period; a longer acting drug would be expected to be more effective. However, a single dose of SP should be able to clear any parasites sequestering in the placenta, which are acquired during the periods when drug levels are too low to provide protection.

Many countries in Africa have scaled up delivery of IPTp with SP substantially, approaching the targets set by the Roll Back Malaria Partnership and WHO. However, IPTp with SP as a malaria control strategy is now challenged from two directions – SP resistance and a decreasing burden of malaria in many, previously highly endemic countries. The ability of an anti-malarial drug to prevent malaria in the face of increasing drug resistance may persist longer than its ability to cure clinical infections [3], but a point will be reached when it can do neither and this point has now been reached in many parts of the malaria endemic world. Which anti-malarial could be used to replace SP? Mefloquine has some of the desirable characteristics, providing a long period of prophylaxis and trials of IPTp with mefloquine conducted in Malawi [10] and Benin [11] have shown that it is effective but side effects are a problem when the drug is used in this way. The combination of SP with amodiaquine (AQ) was also effective, but AQ was not well tolerated [12]. Other combinations being explored are azithromycin combined with SP or CQ [13]. Although azithromycin is a relatively ineffective anti-malarial it is an effective treatment for several sexually transmitted diseases, some of which can cause low birth weight. This property of azithromycin could justify the continuing use of IPT in communities where the risk of malaria is declining. The combination of artesunate and piperaquine has been proposed as another alternative for IPTp but the use of an artemisinin for IPTp does not seem logical because of its short half-life. A new, safe alternative to SP is needed for IPTp.

IPTp has proved to be a valuable tool for the control of malaria in communities with medium or high levels of transmission. However, as the incidence of malaria declines, the benefits of IPTp may be outweighed by the risks and costs. Below a certain level of risk, screening of women who attend an antenatal clinic using either microscopy or a rapid diagnostic test (RDT) and treatment of just those who are positive may be a more rational and cost effective approach than giving anti-malarials to a large number of pregnant women who are not at risk. However, at what level of transmission this transition should take place is not known and needs to be investigated.

## Infants

In areas of very high and perennial malaria transmission, infants account for a substantial proportion of malaria deaths and cases of severe disease. In such epidemiological situations, infants are an important target group for malaria control strategies including chemoprevention. A study of chemoprophylaxis showed that this was an effective way of preventing malaria and anaemia in Tanzanian infants but there was a marked increase in the incidence of both malaria and anaemia in the year after chemoprophylaxis was stopped - rebound malaria [8]. This finding led to the evaluation of a more restricted use of chemoprevention in the first year of life – IPT in infants (IPTi). IPTi takes advantage of the high coverage of routine infant immunization achieved in many malaria endemic countries to administer a course of anti-malarial treatment at the time of vaccination. IPTi has the major advantage that a delivery system is already in place so that costs are largely restricted to those of the drugs. An initial trial of IPTi in Tanzania, in which three doses of SP were given during the first year of life at the time of routine immunization, showed highly encouraging results with an approximately 50% reduction in clinical attacks of malaria and in anaemia [14,15]. These encouraging results led to further evaluation of the potential of this intervention in a number of trials conducted across Africa, which were coordinated by the IPTi Consortium. Combination of results from these studies showed an approximately 30% reduction in the incidence of clinical malaria with a variable impact on the incidence of anaemia and hospital admissions [16]. No significant rebound effect was noted in the combined analysis although there was some suggestion of a rebound in some individual trials. No impact on mortality was detected but none of the trials was powered to detect this. IPTi with SP was well accepted by the community, cost effective and readily implementable[17-19]. On the basis of these findings, both an independent evaluation of IPTi by the Institute of Medicine [20] and a WHO technical review committee recommended that IPTi should be implemented in communities with medium of high levels of malaria transmission and no significant resistance to SP.

Most studies of IPTi have used SP, which has the major advantage that a full treatment course requires only one dose. However, in many of the communities where IPTi might be most effective there is a significant level of SP resistance and an alternative drug is needed. Mefloquine proved highly effective but, as in pregnancy, caused a high incidence of sire effects, especially vomiting [21]. Lapdap, chlorproguanil plus dapsone, was not effective indicating the need for a long-acting drug. Piperaquine has a long action and might be a suitable replacement for SP, but currently is licensed only in combination with dihydroartemisinin which is unlikely to contribute much to IPTi as children who have clinical malaria at the time of administration of IPTi are treated with a full course of the recommended first-line treatment for malaria. A knowledge of the pharmacokinetics of anti-malarial drugs is essential in determining how they can be deployed most rationally for IPTi or IPT in older children (IPTc) [22].

A number of studies have been undertaken recently to model the impact of IPTi on morbidity from malaria in different epidemiological situations [23,24] and its potential impact on drug resistance [25].

IPTi is likely to be most effective in communities where malaria transmission remains high and persists throughout most of the year. As the endemicity of malaria decreases in response to other malaria control interventions, the proportion of cases of severe and uncomplicated malaria in infants falls with the main burden of the infection falling on older children, making implementation of IPTi a less attractive prospect in such communities.

## Children

In communities where the level of malaria transmission is only moderate and in those where malaria transmission is restricted to just a few months of the year, the main burden of severe malaria is in older children who would not be protected by IPTi. Thus, the impact of administering IPT in older children has been explored, largely in countries where malaria transmission takes place during only a few months each year. Most of the Sahelian and sub-Sahelian regions of Africa, which support nearly half of the population of sub-Saharan Africa, falls into the latter category. Seasonal IPT in children (IPTc), which involves giving a long acting anti-malarial or anti-malarial combination at one or two monthly intervals for a few months during the period of peak malaria transmission, is effectively a form of targeted chemoprophylaxis.

Studies conducted in Ghana, Mali, Senegal and The Gambia have shown that IPTc is a highly effective intervention, reducing clinical attacks of malaria by 70% - 90%, and reducing substantially all cause and malaria specific hospital admissions [26-29] . A number of drug combinations have been evaluated for use in IPTc. Because the drug pressure applied by use of IPT in all children under the age of five or ten years is greater than that for IPTp or IPTi , it seems prudent to use a drug combination rather than monotherapy for IPTc and to use a different combination to the one used for first line treatment. A number of drug combinations have been tested for IPTc [30] with most experience having been gained with SP + AQ. This combination has proved highly effective in several studies although in some, but not all, studies vomiting, probably caused by AQ, has been an issue. This could probably be alleviated by preparing a more palatable formulation than crushed tablets or by adjusting the content of AQ tablets to make accurate dosing more readily achieved [31]. Other drugs combinations investigated include artesunate + SP, piperaquine + SP and artesunate + piperaquine.

IPTc has the major disadvantage over IPTi and IPTp that no established system for the delivery of the drug exists in most situations where this approach to malaria control might be applied. However, a number of studies have shown that high levels of coverage can be achieved using community volunteers. Implementing community delivery programmes outside the research situation would be a major challenge but the benefits in terms of lives saved and episodes of malaria prevented would be high if this could be achieved.

## School children

Mass treatment programmes to control intestinal helminths and schistosomiasis are in progress in a number of malaria endemic countries and provide a vehicle through which anti-malarial drugs could be given. A study conducted in Uganda in which children were given a course of SP +AQ once a term showed a substantial reduction in the incidence of anaemia in children who received IPT and an improvement in some aspects of their school performance [32]. Similar results have been obtained in Mali [33]. Whether or not IPT is an appropriate method for control of malaria in schoolchildren is being investigated further but the results of these studies demonstrate that malaria may be a more important cause of ill health and poor performance in school children than had previously been recognized.

## Mass drug administration

The term mass drug administration (MDA) has been used to describe several different approaches to malaria control and is thus confusing. These include administration of anti-malarials on one or more occasions to the whole of a population at risk with the objective of reducing the burden of clinical malaria (thus overlapping with IPT) or with the objective of interrupting transmission, two very different objectives requiring different approaches. Drugs have been given either through mass treatment campaigns or through the fortification of salt with anti-malarials (the Pinotti method). MDA has a bad reputation in many quarters, including WHO, stemming in part from the experience with fortified salt which, as might have been anticipated, facilitated the emergence and spread of resistance by exposing a large proportion of a population at risk of malaria to sub-therapeutic concentrations of a drug over a prolonged period of time [34].

Several studies of MDA, sometimes combined with vector control, were carried out in the 1950s, 1960s and 1970s and showed that MDA, especially if given repeatedly, could reduce parasite prevalence and the incidence of clinical malaria substantially but that this effect was only transitory and MDA rarely interrupts transmission [35]. The results of a large study study conducted in Garki, northern Nigeria, which showed that several rounds of MDA combined with intensive vector control did not interrupt transmission contributed to the loss of interest in MDA as a malaria control tool. However, resurrection of the prospects of malaria elimination has reawakened interest in this approach.

## Chemoprevention and elimination

Several countries, including several in Africa, have made considerable progress in bringing malaria under control during the past few years reawakening interest in the possibility of local or regional elimination. It is now generally accepted that in medium or high transmission areas, the currently deployed malaria control tools – treatment with an artemisinin combination (ACT) and vector control with ITNs and/or IRS - will alone be insufficient to interrupt transmission and that an additional tool will be needed to do this. This might be a vaccine with transmission-blocking properties or it might be a drug given as MDA. Examples of the way in which MDA can contribute to malaria elimination is provided by the successful elimination programme on the island of Aiyetun in Vanuatu [36] and recent success in probable interruption of transmission in an area of Cambodia [37].

If an anti-malarial drug or drug combination is used to assist in interrupting transmission it must have good transmission-blocking properties. Artemisinins are partially effective but subjects treated with an ACT can still transmit malaria [38] so an additional, or more effective, gametocytocidal drug is needed if transmission is to be completely prevented. Currently, the only licensed drug that can completely prevent transmission of *Plasmodium falciparum* is primaquine (*Plasmodium vivax* gametocytes are killed by most anti-malarial drugs) and there are concerns about the safety of this drug when used for MDA in populations where glucose-6-phosphate-dehydrogenase (G6PD) deficiency is common. Tafenoquine, now in clinical trials, has the advantage over primaquine that it has a much longer period of activity, thus providing prophylaxis as well as treatment, but it can also cause haemolysis in G6PD positive subjects. Concerns over the potential toxicity of using primaquine for MDA in communities where G6PD deficiency is common has led to investigation of the alternative approach of mass screening and treatment of just those who are parasite positive. However, the ability of microscopy or current RDTs to detect very low levels of infection, which may still be sufficient to allow transmission [39], makes it uncertain whether this strategy would work as a component of an elimination strategy unless very sensitive molecular techniques could be used.

Previous experience suggests that local or regional elimination of *P. vivax* will be more difficult than elimination of *P. falciparum,* because of the persistence of hypnozoites. If MDA is to be used as a major tool in *P. vivax* elimination campaigns the drug or drug combination used must be able to kill hypnozoites and, currently, the only way that this can be done is with a prolonged course of primaquine. A more effective drug than primaquine, one which can ideally be given as single dose, will be needed if drugs are to play an important role in the elimination of *P. vivax* and *P.ovale* infections.

## Conclusions

Drugs are generally a less satisfactory way of preventing infections than vaccines as they have to be taken regularly over the period for which protection is required. In the case of malaria this might be for life, a challenging prospect. However, in a number of situations, anti-malarials have an important role to play in preventing mortality and morbidity from malaria until a time is reached at which other more readily delivered control measures are effective on their own. Once a high level of control has been achieved, anti-malarials may acquire a new role as an important contributor to local or regional elimination.

## Competing interests

The authors declare that they have no competing interests.
